# Post-weaning blood transcriptomic differences between Yorkshire pigs divergently selected for residual feed intake

**DOI:** 10.1186/s12864-016-2395-x

**Published:** 2016-01-22

**Authors:** Haibo Liu, Yet T. Nguyen, Dan Nettleton, Jack C. M. Dekkers, Christopher K. Tuggle

**Affiliations:** Department of Animal Science, Iowa State University, 2258 Kildee Hall, Ames, IA 50011 USA; Department of Statistics, Iowa State University, 1121 Snedecor Hall, Ames, IA 50011 USA; Institute of Mathematics, Vietnam Academy of Science and Technology, Hanoi, Vietnam; Department of Animal Science, Iowa State University, 239 Kildee Hall, Ames, IA 50011 USA; Department of Animal Science, Iowa State University, 2255 Kildee Hall, Ames, IA 50011 USA

**Keywords:** *Sus scrofa*, Feed efficiency, Residual feed intake, Blood transcriptome, RNA-seq

## Abstract

**Background:**

Improving feed efficiency (FE) of pigs by genetic selection is of economic and environmental significance. An increasingly accepted measure of feed efficiency is residual feed intake (RFI). Currently, the molecular mechanisms underlying RFI are largely unknown. Additionally, to incorporate RFI into animal breeding programs, feed intake must be recorded on individual pigs, which is costly and time-consuming. Thus, convenient and predictive biomarkers for RFI that can be measured at an early age are greatly desired. In this study, we aimed to explore whether differences exist in the global gene expression profiles of peripheral blood of 35 to 42 day-old pigs with extremely low (more efficient) and high RFI (less efficient) values from two lines that were divergently selected for RFI during the grow-finish phase, to use such information to explore the potential molecular basis of RFI differences, and to initiate development of predictive biomarkers for RFI.

**Results:**

We identified 1972 differentially expressed genes (DEGs) (*q* ≤ 0.15) between the low (*n* = 15) and high (*n* = 16) RFI groups of animals by using RNA sequencing technology. We validated 24 of 37 selected DEGs by reverse transcription-quantitative PCR (RT-qPCR) in a joint analysis of 24 (12 per line) of the 31 samples already used for RNA-seq plus 24 (12 per line) novel samples from the same contemporary group of pigs. Using an analysis of the 24 novel samples alone, only nine of the 37 selected DEGs were validated. Genes involved in small molecule biosynthetic process, antigen processing and presentation of peptide antigen via major histocompatibility complex (MHC) class I, and steroid biosynthetic process were overrepresented among DEGs that had higher expression in the low versus high RFI animals. Genes known to function in the proteasome complex or mitochondrion were also significantly enriched among genes with higher expression in the low versus high RFI animals. Alternatively, genes involved in signal transduction, bone mineralization and regulation of phosphorylation were overrepresented among DEGs with lower expression in the low versus high RFI animals. The DEGs significantly overlapped with genes associated with disease, including hyperphagia, eating disorders and mitochondrial diseases (*q* < 1E-05). A weighted gene co-expression network analysis (WGCNA) identified four co-expression modules that were differentially expressed between the low and high RFI groups. Genes involved in lipid metabolism, regulation of bone mineralization, cellular immunity and response to stimulus were overrepresented within the two modules that were most significantly differentially expressed between the low and high RFI groups. We also found five of the DEGs and one of the co-expression modules were significantly associated with the RFI phenotype of individual animals (*q* < 0.05).

**Conclusions:**

The post-weaning blood transcriptome was clearly different between the low and high RFI groups. The identified DEGs suggested potential differences in mitochondrial and proteasomal activities, small molecule biosynthetic process, and signal transduction between the two RFI groups and provided potential new insights into the molecular basis of RFI in pigs, although the observed relationship between the post-weaning blood gene expression and RFI phenotype measured during the grow-finish phase was not strong. DEGs and representative genes in co-expression modules that were associated with RFI phenotype provide a preliminary list for developing predictive biomarkers for RFI in pigs.

**Electronic supplementary material:**

The online version of this article (doi:10.1186/s12864-016-2395-x) contains supplementary material, which is available to authorized users.

## Background

Feed efficiency (FE) is one of the important traits directly related to profitability, productivity, and sustainability in the pork industry [1, 2]. While many non-genetic strategies have been developed to improve feed efficiency of pigs [3], improving feed efficiency by genetic selection can be a sustainable alternative. An increasingly popular measure of FE is residual feed intake (RFI), which is the difference between the actual and expected feed intake of an animal for production and maintenance [4]. Importantly, RFI is moderately heritable and responds well to genetic selection in pigs [5, 6].

Over the last decade, significant efforts have been made to understand the molecular, genetic and physiological basis of RFI in pigs. Researchers have found many interesting differences across multiple tissues between pigs with divergent RFI phenotypes [7, 8]. For example, as compared to high RFI pigs (less efficient), low RFI pigs (more efficient) have altered feeding behaviors [9], slightly lower growth rate [10], less back fat [6, 11, 12], lower protein turnover rate in the muscle [13], altered mitochondrial protein profiles [14, 15], less mitochondrial reactive oxygen species (ROS) production [16], and lower levels of leptin [17, 18] and juvenile IGF-1 in circulating blood [10]. To explore the genetic basis of RFI in pigs, several genome-wide association studies (GWAS) have been conducted [19–22]. Some chromosomal regions tagged by single nucleotide polymorphisms (SNPs) have been found to be associated with RFI, but these associations were not consistent across studies and explained only small portions of the genetic variance for RFI [19–22]. The wide range of differences across multiple tissues between lines of pigs with divergent RFI and the lack of SNPs with major effects on RFI suggest that RFI is a highly polygenic, quantitative trait with multiple tissues contributing to its variation.

Global gene expression profiling technologies have also been used to explore the molecular basis of RFI in pigs. By profiling the transcriptomes of the adipose tissue of two lines of pigs divergently selected for RFI with gene expression microarrays, Lkhagvadorj *et al*. [23] found that genes involved in the lipid metabolic pathway were overrepresented among the differentially expressed genes (DEGs) that had lower expression in low versus high RFI pigs, and genes involved in carbohydrate metabolism and response to stress were overrepresented among the DEGs that had higher expression in low versus high RFI pigs [23]. They also found the leptin-related gene network to be different between the two lines [23]. Recently, Vincent *et al.* [24] and Jing *et al*. [25] profiled the transcriptome of the *longissimus dorsi* (LD) muscle from pigs with divergent RFI by gene expression microarray and RNA-seq, respectively. Using pigs from lines divergently selected for RFI, Vincent *et al.* [24] found genes involved in protein synthesis and glycolysis, and genes associated with mitochondrial energy/oxidative metabolism had higher and lower expression, respectively, in the low versus high RFI line. Using Yorkshire barrows with extreme phenotypes for RFI, Jing *et al*. [25] found that genes involved in glycolysis had lower expression in the low versus high RFI group, while genes involved in muscle proliferation and differentiation had higher expression in the low versus high RFI group. Surprisingly, these two studies shared no DEGs and proposed opposite differences in glycolytic activities in the low versus high RFI pigs. Therefore, in consideration of the complexity of RFI and the inconsistency from study to study, the molecular mechanisms underlying RFI in pigs are still largely unclear.

To incorporate RFI into animal breeding programs for improving feed efficiency, feed intake, body weight gain and back fat depth must be recorded on individual pigs. As it is very expensive and time-consuming to record feed intake on individual animals [5, 6], convenient and predictive biomarkers for RFI that can be measured at an early age are in demand. In cattle, Chen *et al*. [26] successfully used 14 DEGs identified in the liver of Angus bulls that were divergently selected for RFI to classify Angus steers from the same divergent RFI lines, and Al-Husseini *et al*. [27] developed a RFI predictor using 8 of these 14 DEGs and validated it in an independent Angus population. These biomarkers are, however, not very practical because invasive liver biopsies are needed.

The peripheral blood is an informative tissue not only because it carries a variety of cells directly involved in immunity and inflammation, but also because it interacts with every organ and tissue in the body via bioactive circulating factors, such as nutrients, metabolites, cytokines, hormones and exosomal cargoes, which are released from the same or different organs or tissues [28]. These bioactive factors interact with blood cells and thus might modify the gene expression profiles of the blood cells dynamically. Molecular signatures in circulating blood, including gene expression profiles, have been shown to reflect the physiopathological status, growth stage and lifestyle of subjects [28–32]. Due to its easy accessibility and informativeness, blood has become a popular sample (direct or as a surrogate) for disease diagnosis, prediction, prognosis, and biomarker discovery [28, 29]. Interestingly, the concentration of IGF-1 in serum at a young age has been shown to be different between animals with divergent RFI in poultry and livestock, including pigs [10, 17, 33, 34]. Blood cell profiles at early growth stages have also been found to be different between livestock with divergent RFI phenotypes, including pigs [35] and cattle [36]. These results suggest that animals with divergent RFI phenotypes, measured later in life, have early physiological differences in circulating blood that may be reflected in blood gene expression profiles at these early stages.

In this study, our objective was to determine the blood transcriptomic differences between post-weaning pigs from two lines divergently selected for RFI, to explore potential molecular mechanisms underlying RFI in peripheral blood and to develop a list of candidate biomarkers for RFI prediction. We hypothesized that post-weaning expression levels of some genes in whole blood were correlated with RFI phenotype measured during the grow-finish phase. We identified 1972 DEGs with *q* ≤ 0.15 and four co-expression modules that were differentially expressed between the low and high RFI groups. A set of selected DEGs were validated by reverse transcription-quantitative PCR (RT-qPCR). Several interesting biological processes underlying DEGs and differential co-expression modules were suggested. We also identified several candidate biomarkers for RFI.

## Methods

### Animals, blood sample collection and complete blood count (CBC) test

**Fig. 1:**
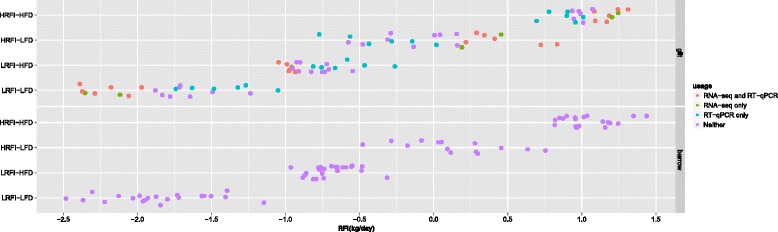
Distribution of RFI values and sample selection for RNA-seq and RT-qPCR assays. Post-weaning blood samples of 16 pigs (8 per diet) with extremely low RFI from gilts of the low RFI line (LRFI) and 16 pigs (8 per diet) with extremely high RFI from gilts of the high RFI line (HRFI) fed the high-fiber, low-energy diet (HFD) or the low-fiber, high-energy diet (LFD) were selected for RNA-seq. One sample in the LRFI-LFD group was excluded from RNA sequencing because of low quality RNA, leaving a total of 31 samples for RNA-seq. The green and red dots represent individuals selected for RNA-seq. Twelve samples from each line by diet combination were selected for RT-qPCR validation of DEGs such that the corresponding RFI phenotypes were representative. The red and blue dots represent the samples selected for RT-qPCR assays. The 24 novel blood samples were selected such that the RFI values of the corresponding animals were roughly evenly distributed across the ranges of RFI not covered by the RFI phenotypes of the 24 animals originally selected for RNA-seq. The distribution of RFI values of barrows from each line by diet combination is also shown for reference

The experimental protocols for this study were approved by the Institutional Animal Care and Use Committee (IACUC) at Iowa State University under permit number 11-1-4996-S. All pigs were from parity 2 of generation nine of the two lines divergently selected for residual feed intake: the low and high RFI lines [6, 19, 35]. Bunter *et al*. found that the IGF-1 concentration in blood measured between 35 to 42 days of age differed between the two lines and were genetically correlated with RFI measured later during the grow-finish phase [10]. So blood samples were collected from the jugular vein into Tempus™ Blood RNA tubes (Life Technologies, Grand Island, NY) for long-term storage at −80 °C from 233 post-weaning piglets of the two lines in this age range. Meanwhile, blood from those animals was also collected into EDTA tubes (BD, Franklin Lakes, NJ) and kept at 4 °C before CBC tests, which were performed on the bleeding day, if possible, or the next morning as described [35, 37]. Differences in CBC profiles between the two lines have been published elsewhere [35]. At 107.0 ± 8.3 (mean ± standard deviation) days of age and 42.2 ± 7.2 kg of body weight (BW), 88 gilts and 78 barrows were randomly assigned to 12 mixed-line, mixed-sex finishing pens with electronic single-space feeders (FIRE, Osborne Industries Inc., Osborne, KS) for feed efficiency test, with 6 pens being randomly assigned to either of two diets: a high-fiber, low-energy diet (HFD) and a low-fiber, high-energy diet (LFD) [38]. The animals on feed efficiency test consisted of 21 barrows and 22 gilts from the low RFI line plus 14 barrows and 23 gilts from the high RFI line fed the HFD, and 23 barrows and 23 gilts from the low RFI line plus 20 barrows and 20 gilts from the high RFI line fed the LFD. All pigs had *ad libitum* access to feed and water. Individual feed intake was real-time recorded, body weight was recorded biweekly, and backfat (BF) depth above the 10th rib and loin muscle area were recorded at the end of the test, when the pigs were 227.0 ± 1.4 days of age and 127.7 ± 8.8 kg of body weight. RFI of individual pigs were calculated as described [19] with modifications and shown in Fig. 1. Briefly, average daily feed intake (ADFI) was estimated by fitting a quadratic polynomial regression model of the daily feed intake from the on-test day to the off-test day on the number of days on test for each pig; and individual average daily gain (ADG) was estimated as the slope from simple linear regression of bi-weekly BW on the number of days on test [6]. A single trait animal model was used to analyze ADFI with adjustments for fixed covariates of metabolic mid-body weight (average body weight during the test period raised to the power of 0.75, MBW), ADG, BF, deviation of the on-test weight from 50 kg, deviation of the off-test weight from 118 kg, and deviation of the on-test age from 90 days, and the random effect of pen. The RFI value for each pig was estimated as the residual of the fitted model (Young J and Dekkers JCM, unpublished). The growth performance and feed intake data of these animals have been published [39].

### RNA extraction and globin depletion

Based on the RFI phenotype of individual pigs, the juvenile blood samples of 32 out of the 88 gilts were selected for RNA-seq. In detail, for the low RFI line, we selected eight samples from animals with extremely low RFI values on the LFD and eight samples from animals with extremely low RFI values on the HFD (designated as low RFI group); while for the high RFI line, we selected eight samples from animals with extremely high RFI values on the LFD and eight samples from animals with extremely high RFI values on the HFD (designated as high RFI group) (Fig. 1). Total RNA extraction of the 32 samples was processed in four batches such that for each batch, two samples were randomly selected from each line by diet combination. Within each batch, the processing order of samples was randomized beforehand and followed in all subsequent procedures where blocking was necessary. The total RNA was extracted from blood samples using preserved blood RNA purification kit I (Norgen Biotek Corp, Thorold, Ontario) by following the kit’s manual. On-column DNA digestion was performed as described using DNase I (Qiagen, Valencia, CA). Globin transcripts (HBB [ENSSSCG00000007978] and HBA [ENSSSCG00000014725]) were depleted by following an RNase H-mediated method [40]. The quantity and integrity of the RNA were determined by using Nanodrop 2000 (Thermo Scientific, Waltham, MA) and Bioanalyzer 2100 (Agilent Technologies, Santa Clara, CA) before and after globin depletion. The efficiency of globin depletion of each sample was checked by conventional RT-qPCR with β-actin (ACTB) and glyceraldehyde 3-phosphate dehydrogenase (GAPDH) as the internal reference genes. The total RNA of one selected blood sample from the low RFI line on the LFD was dropped from this study due to its low RNA quality (original RNA integrity number (RIN) was 7.5, lower than our criterion of RIN ≥ 8.0 before globin depletion). The average RIN for the 31 remaining samples before and after globin depletion were 9.05 ± 0.31 and 8.26 ± 0.30, respectively. Detailed information about the selected samples, including pedigree, RNA quality, batch, and CBC is available in Additional file 1: Table S1.

### RNA-sequencing

Library construction and sequencing were performed by the DNA facility at Iowa State University. Briefly, the RNA-seq libraries were constructed using the Illumina TruSeq RNA Sample Preparation Kit v2 (Illumina, San Diego, CA) according to manufacturer’s instructions. For each sample, poly (A)-containing transcripts was enriched with oligo-dT-coated magnetic beads from 0.7 to 2 μg of total RNA with globin transcript depleted. The enriched RNA was fragmented by heat and reverse transcribed with hexamer random primers. For each sample, adapters with unique barcodes were ligated to the end-polished cDNA fragments. The libraries were linearly amplified by PCR, size selected and quantitated. The individual libraries were diluted to 2 nM and pooled in approximately equimolar amounts according to the processing batches mentioned above with eight libraries per pool, except for one pool of seven libraries. One hundred base paired-end sequencing was run on an Illumina HiSeq2000 platform with one pool per lane on a flow cell.

### Quality control, preprocessing and alignment of RNA-seq reads

Read quality was checked by using FastQC (version 0.10.1) [41]. Adapters and low quality bases were trimmed by running Trimmomatic (version 0.32) [42] in the paired-end mode with the following options, ILLUMINACLIP:adapters.fa:2:30:10:1:true LEADING:3 TRAILING:3 SLIDINGWINDOW:4:15 LEADING:3 TRAILING:3 MINLEN:36, such that the average base quality was not lower than 15 for every sliding window of 4 bases and the minimum length of kept reads was 36 bases. For each set of raw paired-end reads, Trimmomatic outputted a pair of files for the kept paired-end reads, a file for unpaired forward reads (R1), and a file for unpaired backward reads (R2). Trimmed paired-end and unpaired reads were separately aligned to the pig reference genome Sscrofa 10.2 (version 77, Ensembl) using 2-pass rna-STAR (version 2.3.0), using the default settings [43, 44]. Read counts per gene per library were summarized by using featureCounts (version 1.4.4) [45], with the resulting SAM files for uniquely mapped, paired-end and unpaired reads as inputs, separately, and using the pig genome GTF file (version 77, Ensembl) as the genomic annotation reference file. The default settings for other featureCounts options were used [45]. A final table of read counts per gene of the 31 samples (designated as the count table) was generated by summing up the individual count tables for paired-end and unpaired reads. Prior to differential expression analysis, hemoglobin genes (HBA and HBB) and genes with few reads (average read count no larger than eight, or 28 or more zero read counts across the 31 samples) were removed from the count table to obtain a final count table with 12280 genes. This count table was used for the subsequent differential expression analysis and weighted gene co-expression network analysis (WGCNA) after further transformation and adjustment.

### Differential expression analysis

The statistical programming language R (version 3.1.0) was used for all statistical analyses, unless indicated otherwise. Differential expression analysis was carried out by using the R package “QuasiSeq” (version 1.0-4) [46]. For each of the 12280 genes in the final count table, we used QuasiSeq to fit a full generalized linear model with a negative binomial response and a log link that included an upper-quartile normalization offset [43] and the fixed effects of RFI group (low and high RFI groups), diet (low and high fiber diets), batch (sample processing batches 1 to 4), and the linear covariates of RFI phenotype (estimated RFI value),pre_conc (RNA concentration before globin depletion), pre_RIN (RIN before globin depletion), post_conc (RNA concentration after globin depletion), post_RIN (RIN before globin depletion), and concentrations of neutrophils, lymphocytes, monocytes, eosinophils and basophils. Note that, although blood was collected before pigs were fed the different diets, a diet effect was included in our initial full model because diet affected the component traits that were used to estimate RFI and thus the estimation of RFI. Because not all the variables included in the initial full model may be associated with transcript levels, we used a backward variable selection algorithm to identify the most relevant variables [47]. The final model included RFI group, batch, pre_conc, post_RIN, and the concentrations of neutrophils, lymphocytes, monocytes and basophils as independent variables. The default settings for arguments in all function calls were used unless specified otherwise. In the *QL.fit* function, the “method” argument was set to “optim”. The reported *p*-values, *q*-values and log_2_ (fold change) associated with all tests of significance were calculated by using the QLSpline method.

### Weighted gene co-expression network analysis (WGCNA)

Before co-expression analysis using the R package “WGCNA” (version 1.46) [48], the expression levels for the 12280 genes in the count table were adjusted for all independent variables in the final model used for differential expression analysis except RFI group. Briefly, *log*-counts per million (designated as *log-cpm*) were calculated using the *voom* function of the Bioconductor package “limma” (version 3.20.9) with the upper-quartile normalized counts as input [49, 50]. The *lmFit* function was used to fit a linear model with *log-cpm* per gene feature as the responsible variable, and RFI group, batch, and the linear covariates of pre_conc, post_RIN, and concentrations of neutrophils, lymphocytes, monocytes and basophils as independent variables. Effects associated with relevant variables (batch, pre_conc, post_RIN, and the concentration of neutrophils, lymphocytes, monocytes and basophils) were subtracted from the original *log-cpm*, to create adjusted transformed gene expression values. The data matrix consisting of adjusted *log-cpm* per gene for the 31 samples (hereafter called the adjusted transformed gene expression matrix) was used as the input for WGCNA. WGCNA was performed by following tutorial I [51], with slight modifications as needed. In WGCNA, all correlation coefficients between gene pairs were calculated by using Pearson’s method. A soft-thresholding power of seven was used by assuming the topology of the unsigned weighted gene co-expression network was scale-free. The average linkage method was used for all clustering procedures. The function *cutTreeDynamic* was used for identification of modules. Only modules with a minimum of 30 genes were considered. Modules with eigengene correlations no less than 0.75 were merged using the *mergeCloseModules* function with cutHeight = 0.25. The eigengene of a module is the first principal component of the gene expression values of that module and can be considered as a representation of the expression profiles of genes in the module [48]. We then fitted linear regression models with expression levels of the module eigengenes as the response variable and RFI group as the independent variable. The estimated effect of RFI group and the associated *p*-value for the null hypothesis that the RFI group term was not useful in explaining expression of the eigengene of the module were used to quantify the strength and significance of the association between the eigengene of a module and RFI group.

### Hierarchical clustering, generation of heat map, and multi-dimension scaling analysis

The adjusted gene expression matrix was used for hierarchical clustering, heatmap generation, and multi-dimensional scaling (MDS) analyses. Spearman correlation coefficients for gene expression between samples were calculated and 1 minus this correlation coefficient was used as the distance between a pair of samples for both hierarchical clustering and heatmap construction. The Ward method was used in the function *hclust* for hierarchical clustering of the samples. A heatmap was generated with the *heat.map2* function to visualize the DEGs (*q* ≤ 0.05). MDS with the first two dimensions was used to visualize the relationships of samples with each other by using the function *plotMDS* from the Bioconductor package “limma” (version 3.20.9) [50]. For MDS, the distance between each pair of samples was the Euclidian distance between them based on the expression of all 12280 genes.

### Gene ontology term and pathway enrichment analyses

For all GO term and Ingenuity Pathway Analysis (IPA)-based pathway enrichment analyses, unadjusted *p*-values were reported [52]. We used Bioconductor package “topGO” (version 2.16.0) [52] to perform gene ontology (GO) term analysis, including GO biological process (GO-BP), GO molecular function (GO-MF) and GO cellular component (GO-CC). GO terms associated with each gene were downloaded from Ensembl Biomart (version 79). The “classical” algorithm, which treats all GO terms to be independent of each other, and Fisher’s exact test were used to estimate significance of such enrichment using the function *runTest*. For GO term enrichment analysis of DEGs (*q* ≤ 0.15), we analyzed DEGs with higher and lower expression in the low versus high RFI group separately, while for GO term enrichment analysis of WGCNA modules, genes in a whole module were analyzed together. The maximum subset of the 12280 genes that was associated with at least one GO-BP, GO-MF or GO-CC term, respectively, was used as the reference set (also known as background) in the corresponding GO term enrichment analyses. Significantly enriched GO terms associated with more than 10 annotated genes in the pig genome annotation (version 79) were reported.

We performed other enrichment analyses of the DEGs (*q* ≤ 0.15) using Ingenuity Pathway Analysis (IPA, 2015 spring release) and the Integrated Pathway Analysis Database (IPAD) for Systematic enrichment analysis [53]. For IPA-based analysis, 8965 of the 12280 genes could be mapped to IPA identifiers via the gene symbols of pig genes and these genes were used as the reference set. For networks and upstream regulator analysis, both direct and indirect relationships were considered. The options, “all data sources”, “confidence” and “mutation”, were checked. For species, “all mammals” was checked. For tissues and cell lines, only data on tissues and primary cells were considered. The cutoff for the log_2_ (fold change) was set to 0 and the *q*-value cutoff was set to 0.15. All DEGs were analyzed together, with 1488 of 1972 DEGs mapped to IPA identifiers. For IPAD-based analysis of DEGs, the IPAD web server [54] was used. Of the 1972 DEGs (*q* ≤ 0.15), 1536 genes with human gene symbols were analyzed together. The default reference set was used, as the IPAD server does not allow the user to provide a reference set. The raw *p*-values were corrected for multiple testing using the “BH” method [55] for IPAD-based analysis.

### Validation by RT-qPCR

We attempted to validate DEGs between the RFI groups with *q* ≤ 0.15, |log_2_ (fold change)| ≥ 1 and averaged FPKM (fragments per kilobase of exon per million fragments mapped) [56] within either RFI group ≥ 1, which resulted in 46 genes. All pairs of primers corresponding to the 46 DEGs and six internal reference genes were designed and synthesized by the Fluidigm Corporation (Fluidigm, San Francisco, CA), such that the two primers of each pair were separated by exon-exon boundaries and could amplify all isoforms of the target gene if possible (see Additional file 2: Table S2). The efficiency of each primer pair was tested by conventional RT-qPCR on the DNA Engine Opticon 2 system (BioRad, Hercules, California) by using the standard curve method [57] and only primer pairs with amplification efficiency no less than 0.95 were further considered. As for specificity, only primer pairs that gave products with single peaks in melting curve analyses were used for the downstream RT-qPCR assays. This resulted in 37 pairs of primers for DEGs and two for internal reference genes (YWHAZ and RPL32) passing the test (Additional file 2: Table S2). The samples we used for RT-qPCR validation of the DEGs included 24 of the 31 RNA samples that we had sequenced by RNA-seq, and another 24 novel samples from gilts from the same contemporary group of pigs, with 6 samples from each line by diet combination. The 24 novel blood samples were selected such that the RFI phenotypes of the corresponding animals were evenly distributed across the distribution of RFI phenotypes that was not covered by the RFI phenotypes of the 24 animals originally selected for RNA-seq (Fig. 1). Detailed information about the 48 samples used for validation of DEGs is in Additional file 1: Table S1. RNA was extracted from the novel samples as above in three batches, with two samples from each line by diet combination per batch. The concentration and quality of 24 novel RNA samples were determined as above. Total RNA without undergoing globin depletion was used for cDNA synthesis. By following the Fluidigm User Guide for Real-Time PCR Analysis [58], Real Time-qPCR was done on a 48.48 dynamic array chip (Fisher Scientific, Pittsburgh, PA) using the Biomarker HD system (Fluidigm, San Francisco, CA). Data were analyzed with the Fluidigm Real-Time PCR analysis software with the default settings, to obtain raw *C*_*t*_ values. The raw *C*_*t*_ values were corrected for differences in the amount of input RNA by using the geometric mean of the *C*_*t*_ values of the two internal reference genes for the same RNA samples to get – *∆C*_*t*_ = − (*C*_*t*__*gene*_ – *C*_*t*__*reference*_) [59, 60]. Differential expression analysis was performed by fitting linear models with – *∆C*_*t*_ values as the response variables and RFI group, RNA extraction batch, and the linear covariates of the concentrations of neutrophils, lymphocytes, monocytes and basophils as independent variables. With the high RFI group as the reference, the estimated effects on – *∆C*_*t*_ for RFI group were defined as the –*∆∆C*_*t*_ values. The *p*-values associated with the effect of RFI group were adjusted to get *q*-values by using the “BH” method [55]. If the *q*-value of the significance test of the RFI group was less than 0.15, the corresponding gene was considered differentially expressed between the low and high RFI groups by RT-qPCR. Because the amplification efficiencies (see Additional file 2: Table S2) of the primers were close to 1, the fold changes of gene expression between the low and high RFI group were calculated as 2^–*∆∆Ct*^ [59].

### Association analysis of (eigen)gene expression with RFI phenotype

For association analysis of the expression levels of eigengenes of modules identified by WGCNA with RFI phenotype, the eigengene expression levels were first analyzed with a linear model that included RFI group and diet as fixed effects and RFI phenotype as a covariate, along with the two-way and three-way interactions among these three factors. We did not include RNA processing batch, RIN, and concentrations of the blood cell types in the linear model because the eigengene expression levels were calculated based on gene expression values that had already been adjusted for these effects. Gene expression determined by RT-qPCR was also used to identify genes associated with RFI phenotype. The – *∆C*_*t*_ values for the 37 target genes in the 48 samples were calculated as above and analyzed with a linear model that included RFI group, RNA extraction batch and diet as fixed effects, and RFI phenotype and the concentrations of neutrophils, lymphocytes, monocyte and basophils as covariates, along with the two-way and 3-way interactions among RFI group, diet and RFI phenotype. However, none of the interaction terms were significant after correcting for multiple testing with the “BH” method (*q* > 0.15), thus interaction terms were removed from the models. The *p*-values associated with the regression coefficient(s) on the RFI phenotype covariate were adjusted to get *q*-values by the “BH” method [55]. If the *q*-value was less than 0.05, the association between the (eigen)gene and the RFI phenotype was considered significant.

## Results

### Differentially expressed genes between low and high RFI groups

Mauch *et al*. [39] showed that the low RFI line had significantly lower RFI than the high RFI line on the LFD (*p* < 0.007), but the low RFI line only tended to have lower RFI than the high RFI line on the HFD (*p* > 0.05). Thus, to maximize the contrast, we selected blood samples for RNA-seq from gilts of the low RFI line with extremely low RFI values when fed the LFD or the HFD (designated as low RFI group), and from gilts of the high RFI line with extremely high RFI values when fed the LFD or the HFD (designated as low RFI group) (Fig. 1). The metadata, including RNA concentration, RINs before and after globin depletion, RFI phenotype and CBC test results are in Additional file 1: Table S1. The distribution of the RFI values for all gilts of each line by diet combination is in Fig. 1. To increase the power to detect lowly expressed genes in the whole blood transcriptome, we depleted the most highly expressed transcripts, hemoglobin A and B (HBA and HBB), using the RNase H-mediated method [40]. Alignment of RNA-seq reads to the reference genome showed that globin transcripts had been effectively reduced (Additional file 3: Table S3). After globin depletion, on average, only 0.22 ± 0.29 and 1.86 ± 2.70 % of the trimmed reads mapped to the HBA and HBB genes, respectively. The numbers of raw reads, trimmed reads, and mapped reads for each sample are in Additional file 3: Table S3. In summary, 20.4 ± 7.8 million pairs of 100-bp raw reads were sequenced per sample. After trimming, 81.4 ± 8.0 % of raw paired-end reads were kept as paired-end reads, while 17.6 ± 8.0 % of raw forward reads (R1) and 0.27 ± 0.05 % of raw reverse reads (R2) were unpaired reads. 91 ± 1 % of trimmed fragments were mapped to the pig reference genome, with 83.0 ± 1.2 % (mean ± *sd*) of trimmed fragments being uniquely mapped. After removing genes with extremely low expression (See Methods), and HBA and HBB from the count table, we had expression data for 12280 genes for downstream analyses.

Mpetile *et al*. [35] showed that pigs at 35 to 42 days of age from the low RFI line had lower concentrations of lymphocytes, monocytes and basophils than those from the high RFI line in their peripheral blood. Therefore, we decided to account for the concentration of different blood cell types in our analyses so that we could adjust gene expression for these concentration differences. Although the animals were randomly assigned to one of the two diets after the blood samples were collected, we started with a full model that included diet and RFI phenotype (estimated RFI value) and performed backward selection to establish a final model for the following reasons: 1) the samples for RNA-seq were selected based on the RFI phenotype of the animals; 2) the RFI phenotype depended on both line and diet effects; 3) we were interested in identifying genes that might be associated with RFI phenotype. However, after accounting for RFI group (i.e. the low RFI and high RFI groups) and other variables identified via backward selection (batch, pre_conc, post_RIN, and the concentrations of neutrophils, lymphocytes, monocytes and basophils), we found the RFI phenotype, diet, pre_RIN, post_conc and the concentration of eosinophils were not significantly associated with the expression levels of most genes (*p* > 0.05). With our final selected model, we found expression levels of 836, 0, 3, and 42 genes to be significantly associated with the concentrations of neutrophils, lymphocytes, monocytes and basophils, respectively (*q* ≤ 0.05) (Additional file 4: Table S4). However, we were most interested in DEGs whose expression differences were primarily dependent on line differences. Therefore, for all downstream analysis, we only considered DEGs between the low and high RFI groups, while accounting for the effects of relevant variables: batch, post_RIN, and the concentrations of neutrophils, lymphocytes, basophils and monocytes.

**Fig. 2:**
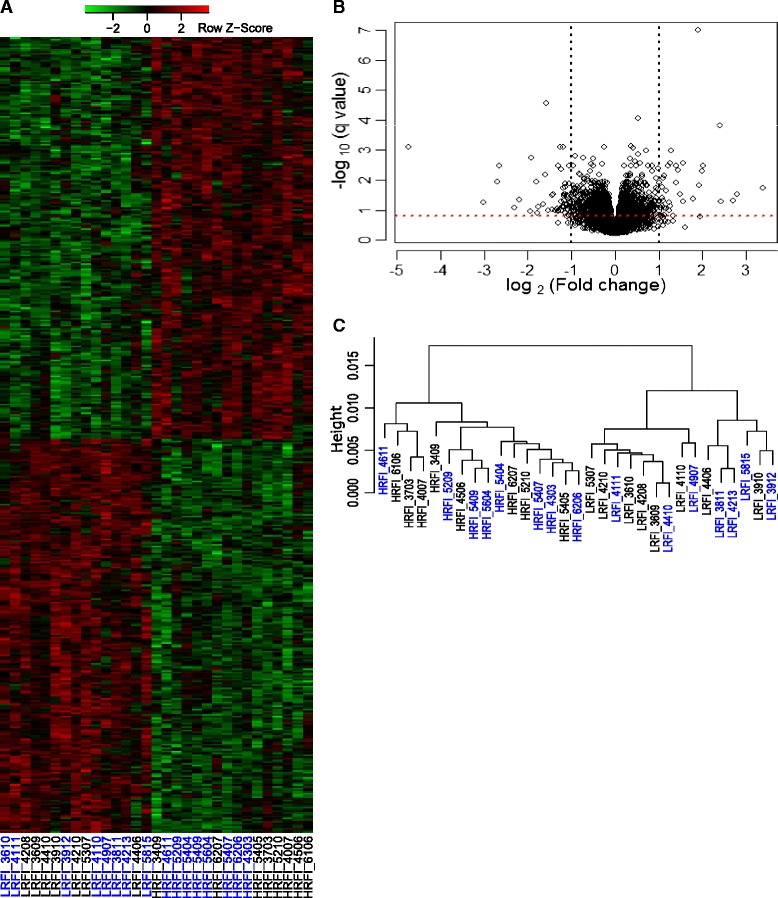
Differentially expressed genes and transcriptomic differences between the low and high RFI groups. **a** Heatmap showing 454 DEGs (*q* ≤ 0.05) between low and high RFI groups identified by RNA-seq. Sample names are designated as RFI line followed by the pig identifier. LRFI, low RFI line; HRFI, high RFI line. Animals with sample names in blue were fed the high-fiber, low-energy diet (LFD), while animals with sample names in black were fed the low-fiber, high-energy diet (HFD). The relative orders of genes and samples were determined by two-way hierarchical clustering based on the adjusted transformed gene expression of the 454 DEGs. The adjusted gene expression was gene-wise standardized to get the *z*-score as displayed. **b** Volcano plot showing the magnitude and significance of differential expression of genes between low and high RFI groups. Black vertical dash lines correspond to │log_2_(fold change)│ = 1, while red horizontal dash line correspond to *q*-value of 0.15. FC, fold change calculated as the ratio of mean gene expression in the low RFI group to mean gene expression in the high RFI group after accounting for the other relevant variables. **c** Hierarchical clustering showing relationship of the 31 RNA-seq samples. The samples were hierarchically clustered by using Ward method with 1 minus Spearman correlation as distance. The Spearman correlations between pairs of samples were calculated based on the adjusted transformed expression of the 12280 genes

The numbers of DEGs between the low and high RFI groups based on different stringencies in terms of *q*-value and fold change cutoffs are in Table 1. A full list of the 1972 DEGs for *q* ≤ 0.15 is in Additional file 4: Table S4. Differential expression of the 454 DEGs with *q* ≤ 0.05 is shown in Fig. 2a. As the volcano plot (Fig. 2b) shows, the fold change between the low and high RFI groups was small for most genes. For a *q*-value cutoff of 0.05, only 50 DEGs had a fold change ≥ 2 or a fold change ≤ 0.5. However, the transcriptomes of the two groups of animals were collectively different, as the 31 samples were grouped into two clusters by RFI group based on hierarchical clustering and MDS analyses (Fig. 2c and Additional file 5: Figure S1).

**Table 1:** The number of differentially expressed genes identified at different *q*-value and fold change cutoffs

Cutoff of *q*-value	Cutoff of fold change		
	│log_2_(FC)│ > 0 ^a^	│log_2_(FC)│ ≥ log_2_ 1.5	│log_2_(FC)│ ≥ 1
*q* ≤ 0.05	454	140	50
*q* ≤ 0.1	1185	266	80
*q* ≤ 0.15	1972	344	93

^a^FC, fold change calculated as the ratio of mean gene expression in the low RFI group to mean gene expression in the high RFI group after accounting for the other relevant variables (see Methods)

### Annotation of differentially expressed genes by GO term and pathway enrichment analyses

To understand the biological differences between RFI groups based on the differential expression analyses, we carried out GO term analysis of DEGs using a less stringent cutoff of *q*-value (*q* ≤ 0.15) such that we could detect a broader group of significant GO terms. These analyses were conducted separately for DEGs with higher and lower expression in the low versus high RFI group. GO-BP term analysis showed that genes involved in small molecule biosynthesis, carboxylic acid biosynthesis, organic acid biosynthesis, steroid biosynthesis, antigen processing and presentation of peptide antigen via MHC (major histocompatibility complex) class I, and organic hydroxy compound biosynthesis were overrepresented among DEGs with higher expression in the low versus high RFI group (*p* < 0.001, Table 2). Among the DEGs with lower expression in the low RFI group, genes involved in signal transduction, bone mineralization, regulation of phosphorylation, and phosphorylation were overrepresented (*p* < 9E-05). Interestingly, GO-CC term analysis showed that genes functioning in the proteasome complex or the mitochondrion were enriched among the DEGs with higher expression in the low versus high RFI group (*p* < 5E-06).

**Table 2:** GO terms overrepresented among differentially expressed genes

ID	Domain	Term	Annotated^a^	Significant^b^	Expected^c^	*p-*value
GO terms overrepresented among DEGs with higher expression in the low RFI group than in the high RFI group						
GO:0044283	BP	Small molecule biosynthetic process	170	33	13.77	1.70E-06
GO:0046394	BP	Carboxylic acid biosynthetic process	113	22	9.15	8.70E-05
GO:0016053	BP	Organic acid biosynthetic process	113	22	9.15	8.70E-05
GO:0006694	BP	Steroid biosynthetic process	44	12	3.56	0.00014
GO:0046165	BP	Alcohol biosynthetic process	48	12	3.89	0.00033
GO:0002474	BP	Antigen processing and presentation of peptide antigen via MHC class I	20	7	1.62	0.00067
GO:1901617	BP	Organic hydroxy compound biosynthetic process	60	13	4.86	0.00084
GO:0004298	MF	Threonine-type endopeptidase activity	15	9	1.18	3.50E-07
GO:0005839	CC	Proteasome core complex	15	9	1.23	5.10E-07
GO:0000502	CC	Proteasome complex	39	14	3.2	1.20E-06
GO:0005739	CC	Mitochondrion	955	116	78.35	4.60E-06
GO:0005689	CC	U12-type spliceosomal complex	17	8	1.39	2.50E-05
GO:0043227	CC	Membrane-bounded organelle	5518	491	452.73	0.00039
GO:0032991	CC	Macromolecular complex	2416	237	198.23	0.00041
GO terms overrepresented among DEGs with lower expression in the low RFI group than in the high RFI group						
GO:0065007	BP	Biological regulation	4577	285	237.14	1.10E-06
GO:0050789	BP	Regulation of biological process	4395	272	227.71	7.00E-06
GO:0007165	BP	Signal transduction	2117	149	109.68	9.70E-06
GO:0030282	BP	Bone mineralization	45	11	2.33	1.30E-05
GO:0023052	BP	Signaling	2276	156	117.92	2.50E-05
GO:0044700	BP	Single organism signaling	2276	156	117.92	2.50E-05
GO:0007166	BP	Cell surface receptor signaling pathway	979	79	50.72	2.90E-05
GO:0031214	BP	Biomineral tissue development	49	11	2.54	3.10E-05
GO:0050794	BP	Regulation of cellular process	4126	254	213.77	4.40E-05
GO:0007154	BP	Cell communication	2328	157	120.61	5.70E-05
GO:0042325	BP	Regulation of phosphorylation	525	48	27.2	7.10E-05
GO:0016310	BP	Phosphorylation	1024	80	53.05	8.10E-05
GO:0044763	BP	Single-organism cellular process	5419	315	280.76	0.00021
GO:0018212	BP	Peptidyl-tyrosine modification	125	17	6.48	0.00023
GO:0001932	BP	Regulation of protein phosphorylation	407	38	21.09	0.00027
GO:0006468	BP	Protein phosphorylation	774	62	40.1	0.0003
GO:0051716	BP	Cellular response to stimulus	2670	171	138.33	0.00041
GO:0060070	BP	Canonical Wnt signaling pathway	86	13	4.46	0.00044
GO:0016477	BP	Cell migration	405	37	20.98	0.0005
GO:0051239	BP	Regulation of multicellular organismal process	841	65	43.57	0.00056
GO:0018108	BP	Peptidyl-tyrosine phosphorylation	123	16	6.37	0.00058
GO:2000026	BP	Regulation of multicellular organismal development	526	44	27.25	0.00099
GO:0040011	BP	Locomotion	511	43	26.48	0.00101
GO:0060089	MF	Molecular transducer activity	409	41	21.42	4.30E-05
GO:0044459	CC	Plasma membrane part	537	45	27.48	0.00063

^a^Number of genes detected in the blood and associated with a given GO term

^b^Number of DEGs associated with a given GO term

^c^Expected number of DEGs associated with a given GO term

In addition, we conducted pathway enrichment analyses of the DEGs (*q* ≤ 0.15) by assuming pig genes have similar biological functions as their human orthologs. Detailed results from these IPA-based analyses are in Additional file 6: Table S5. In summary, IPA-based analysis suggested genes functioning in several canonical pathways tended to be enriched among the DEGs, including p53 signaling (*p* =2.18E-03), T cell receptor signaling (*p* = 1.25E-02), antigen presentation (*p* = 1.63E-02), IL-15 signaling (*p* = 2.16E-02) and IL-9 signaling (*p* = 2.21E-02). But after correcting for multiple testing, none of these pathways were significantly enriched among the DEGs (*q* > 0.1). In addition, IPA-based analyses suggested that activities of genes involved in regulating the quantity of T lymphocytes (*p* = 7.46E-05), consumption of oxygen (*p* = 1.33E-04), insulin resistance (*p* = 1.98E-04), cell survival (*p* = 4.67E-04), glucose metabolism and transport (*p* < 5.64E-03), respiration of mitochondrion (*p* = 5.74E-03) and body mass index (*p* = 1.03E-02) were different between the two RFI groups. IPA-based analysis also suggested several potential upstream regulators of the DEGs, including IL15, which had a higher expression in the low versus high RFI group (*p* < 3.6E-03). The inferred regulatory network for IL15 is shown in Additional file 7: Figure S2.

Using the Integrated Pathway Analysis Database (IPAD) for Systematic Enrichment Analysis [53], we found genes involved in the immune system (pathway ID: 168256) (*q* = 0.013) and metabolism (pathway ID: 1430728) (*q* = 0.066) were enriched among the DEGs. IPAD-based disease-associated gene enrichment analysis suggested genes involved in several diseases were overrepresented among DEGs (*q* < 1E-05), including taste disorder, eating disorder, anorexia, hyperphagia, obesity, insulin resistance, mitochondrial diseases and lymphocytosis. Details are in Additional file 8: Table S6. This is consistent with the phenotypic differences in feeding behavior [9], body composition [12], and growth rate [10] that have been observed between the two RFI lines.

### Validation of DEGs

**Fig. 3:**
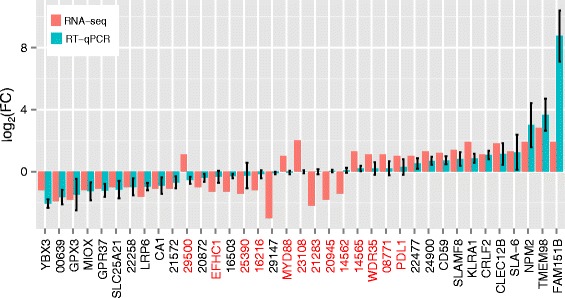
Validation of DEGs by RT-qPCR. 24 of the 37 selected DEGs between low and high RFI groups were confirmed by RT-qPCR when using the 48 samples, 24 of which were used for RNA-seq and another 24 of which were novel, as shown in this figure (*q* ≤ 0.15); 22 of the 37 selected DEGs were confirmed by RT-qPCR when using the 24 samples that were used for RNA-seq (*q* ≤ 0.15); but only 9 of the 37 selected DEGs were validated by RT-qPCR when using the 24 novel samples (*q* ≤ 0.15) (Addition file 9: Table S7). For comparison, the log_2_ (fold change) of these genes determined by RNA-seq were also displayed. Genes were ordered based on their log_2_ (fold change) as determined by RT-qPCR for display. Error bars for RT-qPCR assays show the standard errors of mean log_2_ (fold change). DEGs not confirmed by RT-qPCR are labeled in red. Genes without corresponding human orthologs are labeled with the last 5 digits of their Ensembl gene IDs, with common prefix “ENSSSCG000000” omitted for simplicity. For example, the ID for “29500” should be ENSSSCG00000029500

We used RT-qPCR to validate the differential expression of 37 DEGs that were selected based on average expression levels within RFI group (FPKM > 1), differential expression levels between RFI groups (|log_2_(fold change)| ≥ 1 and *q* ≤ 0.15) and primer performance. To test whether the expression differences we detected were due to differences between the lines, rather than due to the comparison of pigs of the low RFI group to pigs of the high RFI group, we selected another 24 samples from the same parity of the 9th generation of the two RFI lines. These 24 samples were selected such that the RFI values of the corresponding animals were roughly evenly distributed across the ranges of RFI values for each line by diet combination, which were not covered by the RFI values of the 24 animals originally selected for RNA-seq (Fig. 1). The RNA from these new samples as well as the 24 samples used for RNAseq analyses were used in RT-qPCR analyses. Data from the original 24 samples and the new 24 samples were analyzed independently as well as jointly. A total of 24 of the 37 DEGs were confirmed in the joint analysis of all 48 samples (*q* < 0.15, Fig. 3), while 22 of the 37 DEGs were confirmed when analyzing the original 24 samples (*q* < 0.15). However, only 9 of the 37 DEGs were validated in the analysis of the novel 24 samples (*q* < 0.15, Additional file 9: Table S7).

Based on the RT-qPCR results of the 48 samples, we found the expression of 5 of the 37 DEGs (determined by RNA-seq) to be significantly associated with RFI phenotype (*q* < 0.05). They were LRP6 (low density lipoprotein receptor-related protein 6), ENSSSCG00000024900 (T-cell receptor beta chain), ENSSSCG00000008771, PDL1 (CD274 molecule) and ENSSSCG00000020945 (Additional file 10: Table S8).

### Co-expressed gene modules associated with RFI

**Fig. 4:**
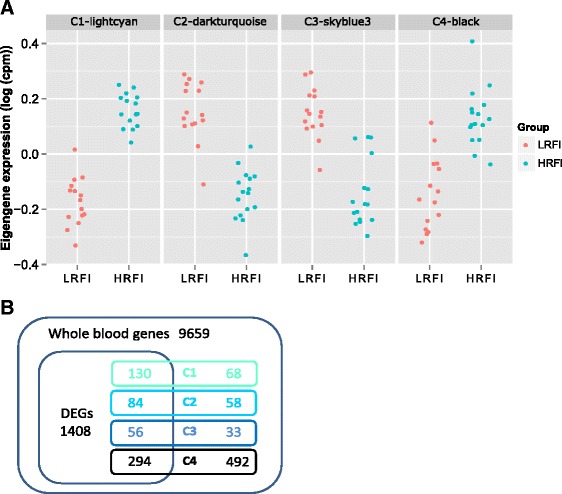
Co-expression modules differentially expressed between the low and high RFI group. **a** Distribution of eigengenes of modules highly associated with RFI groups. **b** Venn diagram showing overlapping between module genes and DEGs

Since conventional differential expression analysis considers each gene independently and suffers from loss of power due to correction for multiple testing, we performed weighted gene co-expression gene network analysis (WGCNA) [48] based on the adjusted gene expression matrix (see Methods) to identify modules (groups of co-expressed genes) differentially expressed between the RFI groups and associated with RFI phenotype. We identified four modules, designated as C1-lightcyan, C2-darkturqoise, C3-skyblue3, and C4-black, whose eigengene expression levels were significantly differentially expressed between the low and high RFI groups (Table 3 and Additional file 11: Table S9). The eigengene of a module is a weighted average of the expression profiles of genes in the module, calculated as the first principal component score. The expression levels of the eigengenes for the 31 samples are shown in Fig. 4a. Among the genes in module C1-lightcyan, those involved in lipid metabolism-related biological processes, such as lipid metabolic process, lipid biosynthesis and steroid biosynthesis were overrepresented (*p* < 0.006). In module C2-darkturqoise, genes involved in biological processes related to bone mineralization, immunity and stress response and lipid metabolism were overrepresented (Additional file 12: Table S10). We also found that DEGs identified by RNA-seq (*q* ≤ 0.15) significantly overlapped with each of the four differentially expressed modules (Fig. 4b), which suggests that the modules identified by WGCNA are not computational artifacts as they were enriched for the DEGs between the RFI lines. In addition, we found that the expression levels of the eigengenes of modules C3-skyblue3 were significantly associated with RFI phenotype (*q* < 0.05) (Additional file 13: Table S11).

**Table 3:** Summary of WGCNA modules differentially expressed between the low and high RFI groups

ID	Size	Reg. coef.^a^	*p*-value	Adjusted R^2^
C1_ lightcyan	198	−0.33	4.7E-13	0.83
C2_darkturquoise	142	0.30	2.9E-09	0.70
C3_ skyblue3	89	0.29	2.7E-08	0.65
C4_ black	786	−0.28	3.9E-07	0.58

^a^Reg. coef., regression coefficient estimated by regressing the expression level of the eigengene of a module on RFI groups, with the high RFI group as the reference

## Discussion

### Small but significant blood transcriptomic differences between the low and high RFI groups

By transcriptomic analyses, we identified 454, 1185 and 1972 DEGs between the low and high RFI groups at *q*-value cutoffs of 0.05, 0.1 and 0.15, respectively. We validated 24 of the 37 selected DEGs by RT-qPCR in a joint analysis of 48 samples: 24 of the 31 samples used for RNA-seq plus 24 novel samples from the same population (Fig. 1, Fig. 3 and Additional file 9: Table S7). We validated 22 of the 37 selected DEGs by RT-qPCR when using 24 of the 31 samples that were used for RNA-seq, but only 9 of the 37 selected DEGs were validated when using only the 24 novel samples (Fig. 1 and Additional file 9: Table S7). Our validation rate by RT-qPCR of DEGs detected by RNA-seq was low compared to the high validation rate reported by Wang *et al*. [61], especially when based on the 24 novel samples. There are several possible reasons for failure to validate some of the 37 DEGs that were detected by RNA-seq by RT-qPCR in this study. First, some of the selected DEGs could be false positives, especially as we selected DEGs with *q* ≤ 0.15 for validation, although we did not detect an obvious association between the validation rate and the *q* values for the DEGs. Second, the primers used for RT-qPCR maybe did not quantify all isoforms that were measured by RNA-seq. Although the primers were designed to amplify all isoforms, these designs were based on poorly annotated gene models: most of the 37 genes only have one isoform in the Ensembl pig genome annotation (Additional file 2: Table S2). Third, the 24 novel samples used for RT-qPCR were not the exact biological replicates of the 24 samples used for RNA-seq given their different RFI phenotype. In addition, as observed in other differential expression analyses between low and high RFI groups conducted in poultry, pigs and cattle [23, 62–65], the magnitude of the differential expression in terms of fold change was generally small for most genes. This could be because the experimental units for the low and high RFI groups were genetically heterogeneous within each group, which cancelled out some differences between extreme individuals of the two groups. In addition, the experiments were conducted without strong external stimuli, thus the expression levels of genes were in their normal physiological range. It was also possible that the differences in gene expression in blood at the post-weaning stage were not as dramatic as during the grow-finish phase when feed efficiency was tested. However, it is possible that small changes in the transcript abundance of some genes involved in metabolism, transcription regulation, and signal transduction, could have significant effects on RFI phenotype. As our differential analysis indicated, a significant portion of genes involved in several biosynthetic processes, signal transduction, and regulation of phosphorylation were differentially expressed between the low and high RFI groups. Lastly, we cannot exclude the possibility that there is a weak or indirect relationship between early blood gene expression and later RFI phenotype. Nevertheless, based on the adjusted global gene expression profiles of all samples, hierarchical clustering and MDS analyses revealed that the post-weaning blood transcriptome was different between the two RFI groups.

### Potential relationship of the DEGs and differentially co-expressed gene modules in blood with RFI

Although we did not attempt to validate most of the DEGs or the differentially co-expressed gene modules between the two RFI groups due to the generally small fold changes, we found some interesting biological processes and cellular components underlying them, which suggested their potential relationships with RFI phenotype. GO term enrichment analysis of DEGs suggested that genes involved in small molecule (including organic acid, carboxylic acid, and alcohol) biosynthesis, antigen processing and presentation of peptide antigen via MHC class I, and steroid biosynthesis were enriched among DEGs with higher expression in the low versus high RFI group. DEGs involved in small molecular biosynthesis included FADS1, FADS2, and ELOVL3. FADS1 and FADS2 are important genes in regulating the synthesis of polyunsaturated fatty acids, which have pleiotropic influences on health and diseases by functioning in several pathways, including metabolism and immunity [66]. Of note, FADS2 also had a higher expression level in the liver in the low RFI versus high RFI group of Nelore steers [62]. ELOVL3 encodes one of the rate-limiting enzymes in elongation of very long chain fatty acids (with more than 17 carbon atoms). In mice, a proposed physiological function of ELOVL3 is to maintain lipid homeostasis by replenishing the intracellular pool of triacylglycerol [67]. MAT2B, is one of the genes associated with steroid biosynthesis but had lower expression in the low versus high RFI group. It encodes a regulatory subunit of methionine adenosyltransferase, MAT II, which catalyzes the synthesis of S-adenosyl methionine (SAM). SAM is a key methyl donor in transmethylation reaction and polyamine biosynthesis and also functions as a cofactor in key metabolic pathways. Down-regulation of MATIIB expression causes a 6 to10-fold increase in intracellular SAM levels [68]. Taken together, the expression difference of genes involved in small molecule biosynthesis and steroid biosynthesis might have significant effects on overall metabolism, immunity and beyond.

Seven of the 20 genes associated with GO term GO:0002474 (antigen processing and presentation of peptide antigen via MHC class I) had higher expression in the low RFI group. These included SLA-3 and TAP1. It has been shown that in post-weaning pigs resistant to *Escherichia coli* F18, SLA-3 has higher expression level than in susceptible pigs [69]. TAP1 transports antigens from the cytoplasm to the endoplasmic reticulum for loading antigen peptide onto MHC class I molecules [70]. In addition, we found 14 of the 15 DEGs associated with GO term GO:0000502 (proteasome complex) to have higher expression in the low versus high RFI group. The only exception was PSMD5, which had lower expression in the low versus high RFI group. It has been shown that overexpression of PSMD5 inhibits assembly and activity of 26S proteasome [71], which is an important component in processing and presenting intracellular peptide antigen via MHC class I to CD8^+^ T cells. Antigen presenting cells (APC) have both constitutive proteasomes and immunoproteasomes, both of which are important for antigen peptide processing [72]. Notably, an immunoproteasome subunit PSMB8 (also known as β_5i_) had the largest difference in expression level among the 15 DEGs that function in the proteasome complex. Given the increased expression levels of non-inhibitory proteasomal components in blood of the low RFI group, it is likely that proteasomal activity is higher in blood from the low versus high RFI group. Taken together, the expression differences of genes involved in antigen processing and peptide antigen presentation suggest that low RFI animals might have a more active system of antigen peptide processing and presentation, which might improve the robustness of their immune system of the low RFI animals. Consistent with this speculation, Dunkelberger *et al*. [73] recently showed that the low RFI line was less affected by experimental infection with porcine reproductive and respiratory syndrome virus (PRRSv), which mainly infects mature macrophages, derivatives of monocytes. In view of the lower concentration of monocytes in the low RFI line, the putative higher activity of antigen processing and peptide antigen presentation might lead to an increased efficiency of innate immunity in the low RFI pigs. It would be interesting to test the activity of the blood proteasome in more detail in these lines for a role in feed efficiency.

Another interesting finding was that genes functioning in the mitochondrion were significantly overrepresented among DEGs with higher expression in the low versus high RFI group. Of the 955 blood genes associated with GO-CC term mitochondrion (GO:0005739), 116 were DEGs with higher expression in the low versus high RFI group. Mitochondria play important roles in many biological processes, including fatty acid metabolism, amino acid metabolism, and steroidogenesis [74]. Importantly, mitochondrial function has been associated with feed efficiency in poultry, cattle and lambs [75, 76]. As well, it has been shown that the mitochondrial proteome profiles are different between these low and high RFI lines of pigs and the mitochondria from the liver and LD muscle of the low RFI pigs produced less ROS [14–16]. Interestingly, we found that four genes involved in detoxification of ROS, GPX3, SOD1 and CAT, had higher expression in the low versus high RFI group. Another interesting gene that functions in mitochondria is G0S2, which is a noncompetitive inhibitor of adipose lipase, a rate-limiting lipase of triglyceride hydrolysis [77]. G0S2 had higher expression in the low versus high RFI group. It has been shown that G0S2 can inhibit ROS production in endothelial cells [78]. In addition, we found higher expression in blood from the low RFI group for genes that encode 14 mitochondrial ribosomal proteins (MRPL3, 9, 10, 11, 18, 20, 24, 38, 40, 46; MRPS10, 11,15, 27), nine components of mitochondrial complex I (NDUFA4, 8; NDUFB3, 7, 9; NDUFS2, 7; NDUFAB1, NDUFAF3), one component of complex II (SDHA), cytochrome c (CYCS) and four components of complex IV (COA3, COA6, COX5B, COX7C), two components of the complex V (ATP5D, ATP5G3), four key enzymes of the TCA cycle (ACO1, IDH2, IDH3A, SDHA, SUCLG1), four enzymes involved in mitochondrial DNA repair (APEX2, FEN1, OGG1, UNG), and two enzymes involved in nucleotide metabolism (DUT, MTHFD1), and three translocases of the inner/outer membranes (TIMM50, TOMM7, TOMM40). Taken together, it is reasonable to speculate that the low RFI group has more efficient mitochondria and can more efficiently handle oxidative stress.

Among the 1972 DEGs (*q* ≤ 0.15), 288 genes were associated with GO:0007165 (signal transduction), and genes involved in signal transduction were significantly enriched among DEGs with lower expression in the low versus high RFI group. DEGs associated with signal transduction were mainly cytokines, receptors and kinases. Signal transduction-related genes with much lower expression in the low versus high RFI group included LRP6, WNT10B, and FZD6. LRP6 belongs to the low-density lipoprotein receptor family and plays a key role in lipoprotein endocytosis and as a co-receptor in Wnt/β–catenin signaling. LRP6 is also involved in regulating lipid homeostasis and body fat mass [79], while LRP6, WNT10B and FZD6 can function together in the Wnt/β-catenin signaling pathway. Besides playing important roles in bone metabolism, the Wnt/β-catenin signaling pathway also takes part in glycolysis and regulates mitochondrial physiology and insulin sensitivity and is thus linked to metabolic diseases [80, 81]. On the other hand, we found a few genes involved in signal transduction but with much higher expression in the low versus high RFI group, such as IL15 and IRS1. IL15 plays important roles as a pleiotropic cytokine in innate and adaptive immunity [82]. IRS1 is an important player in both the insulin signaling and the IGF-1 signaling pathways and IGF-1 is an important anabolic hormone, playing a key role in growth. Note that juveniles from the low RFI line have been shown to have lower serum concentration of IGF-1 and the low RFI line grows only slightly slower than the high RFI line. The higher expression of IRS1 might partially compensate for the lower level of IGF-1 in the low RFI line. It is interesting to investigate the expression and activity of IRS1 in other tissues of the two lines, such as the muscle. The differential expression of many genes associated with signal transduction might be related to differences in metabolism and immune response between the RFI lines. Lastly, we found four co-expression modules to be differentially expressed between the RFI groups, and these modules shared a significant portion of their genes with the list of DEGs. The eigengene expression of module C1-lightcyan was lower in the low RFI group than in the high RFI group. The top highly connected genes in this module were MAT2B, CPT1A, and LRP6. These genes were also differentially expressed between the low and high RFI groups. Since we have already discussed MAT2B and LRP6, we focus on CPT1A herein. CPT1A had lower expression in the low versus high RFI group and plays an important role in importing long chain fatty acids into mitochondria, by catalyzing the primary step of mitochondrial fatty acid oxidation [83]. Inhibition of hypothalamic CPT1A has been shown to decrease feed intake and glucose production in rats [84]. Thus it will be interesting to investigate its expression in other tissues including the hypothalamus. In addition, GO-BP term analysis showed that genes involved in lipid metabolism-related processes were enriched in module C1-lightcyan. Because low RFI pigs have less body fat and are leaner [12] and that deposition of energy as fat costs more energy than as protein [85], genes in this module might be relevant to differences in RFI.

### Potential predictive biomarkers for RFI

DEGs whose expression levels are associated with the phenotype of interest, as well as genes whose expression profiles are similar to the eigengenes of co-expression modules associated with the phenotype are often considered as good candidate biomarkers for the phenotype. Based on our association tests, the best candidate biomarkers included LRP6, ENSSSCG00000024900, ENSSSCG00000008771, PDL1 and ENSSSCG00000024905. LRP6 and ENSSSCG00000024900 were ones of the DEGs between the two RFI groups and ones of the highly connected, eigengene-like genes in modules C1-lightcyan and C2-darkturqoise, respectively (Additional file 11: Table S9). Notably, we only found a very few genes whose expression levels were significantly associated with RFI phenotype, which might be because the estimated RFI values were residuals which represented both random errors and true feed efficiency and thus were not accurate, or because there were not many genes whose post-weaning expression levels in blood were well associated with the RFI values measured during the grow-finish phase in blood, or because there may be little or no relationship of blood gene expression in blood and RFI phenotype regardless of age.

## Conclusions

As far as we know, this is the first study to explore transcriptomic differences in blood between pig lines with divergent RFI by RNA-seq. We found that the blood transcriptome was clearly different between the low and high RFI groups, although only a small number of genes showed large fold changes of expression between the two groups. The two RFI groups may be different in mitochondrial and proteasomal activities, small molecule biosynthetic process, and signal transduction. These blood transcriptomic differences may be related to the difference in feed efficiency between these two groups, although the observed relationships of post-weaning blood gene expression with RFI phenotype measured during the grow-finish phase were not strong. The top candidate biomarkers for predicting RFI included LRP6, ENSSSCG00000024900, ENSSSCG00000008771, PDL1 and ENSSSCG00000024905.

### Availability of supporting data

The RNA-seq data sets supporting the results of this article is available in ArrayExpress under accession number: [ E-MTAB-4179; https://www.ebi.ac.uk/arrayexpress/].

## Additional files

Additional file 1: Table S1. Meta data for RNA-seq and RT-qPCR assays. (XLSX 20 kb) Additional file 2: Table S2. Primers used to validate differentially expressed genes by RT-qPCR. (XLSX 17 kb) Additional file 3: Table S3. Statistical summary of raw, trimmed, mapped and residual globin reads. (XLSX 17 kb) Additional file 4: Table S4. Differentially expressed genes identified by RNA-seq. (XLSX 5306 kb) Additional file 5: Figure S1. MDS plot showing relationships of RNA-seq samples. (PDF 31 kb) Additional file 6: Table S5. Summary of Ingenuity IPA-based pathway analyses. (XLSX 13 kb) Additional file 7: Figure S2. Subnetwork consisting of differentially expressed genes regulated by the inferred upstream regulator IL-15. Nodes were colored based on the log ratio of averaged expression levels of genes between groups, with up-regulated genes in red and down-regulated genes in green. (PDF 86 kb) Additional file 8: Table S6. Summary of IPAD-based pathway and disease enrichment analyses. (XLSX 59 kb) Additional file 9: Table S7. Validation of differentially expressed genes by RT-qPCR assays. (XLSX 18 kb) Additional file 10: Table S8. Association of gene expression with RFI phenotype. (XLSX 12 kb) Additional file 11: Table S9. Differentially expressed WGCNA modules between the RFI groups. (XLSX 307 kb) Additional file 12: Table S10. GO-BP terms enriched in the WGCNA modules. (XLSX 15 kb) Additional file 13: Table S11. Association of module eigengenes with RFI phenotype. (XLSX 10 kb)

## Abbreviations

ADFI: Average daily feed intake
ADG: Average daily gain
APC: Antigen presenting cells
BH: Benjamini–Hochberg
BW: Body weight
CBC: Complete blood count
cpm: count per million mapped reads
DEG: Differentially expressed gene
FC: Fold change
FE: Feed efficiency
FIRE: Feed intake recording equipment
FPKM: Fragments per kilobase of exon per million fragments mapped
GO-BP: CC and MF, gene ontology-biological process, cellular component, and molecular function
GWAS: Genome-wide association study
HFD: High-fiber, low-energy diet
HRFI: High RFI
IPA: Ingenuity pathway analysis
IPAD: Integrated pathway analysis database
LD: *Longissimus dorsi*
LFD: Low-fiber, high-energy diet
LRFI: Low RFI
MBW: Metabolic mid-body weight
MDS: Multi-dimensional scaling
MHC: Major histocompatibility complex
RFI: Residual feed intake
RIN: RNA integrity number
RNA-seq: RNA-sequencing
ROS: Reactive oxygen species
SNP: Single nucleotide polymorphism
WGCNA: Weighted gene co-expression network analysis
