# Supplementary figures and images for: Post-weaning blood transcriptomic differences between Yorkshire pigs divergently selected for residual feed intake
Source: BMC Genomics. 2016 Jan 22;17:73. doi: 10.1186/s12864-016-2395-x (PMC4724083; doi:10.1186/s12864-016-2395-x)

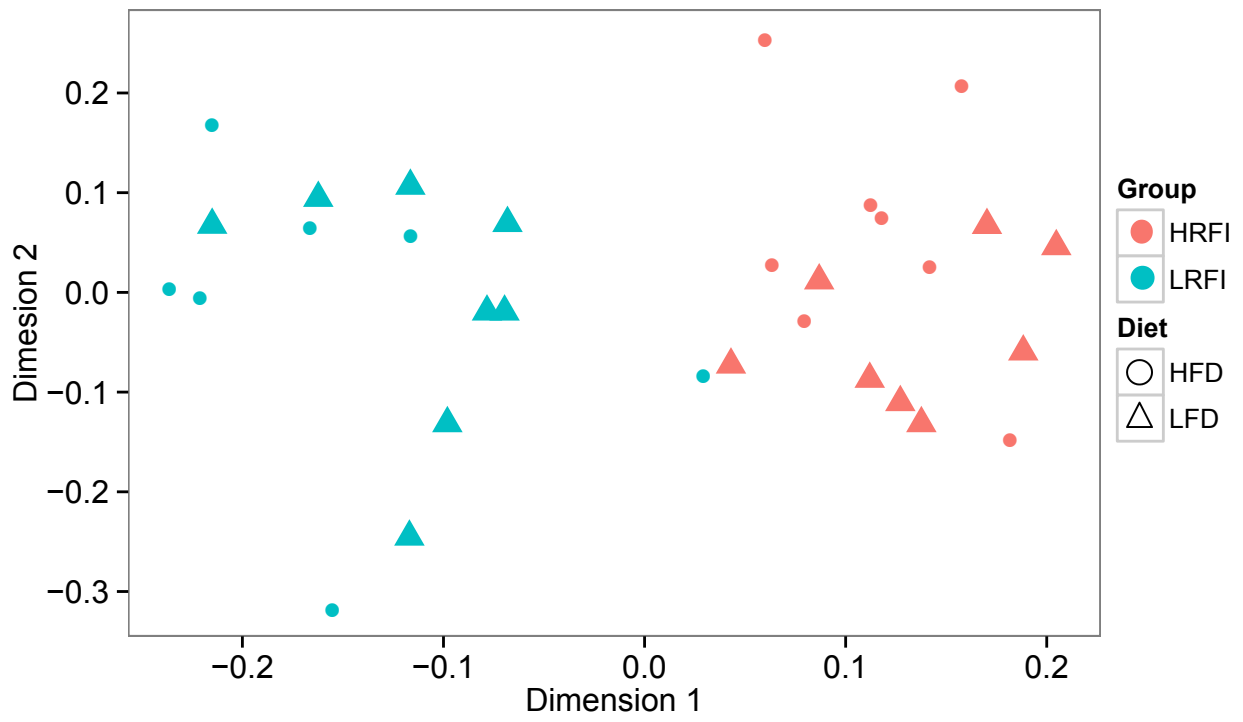

Supplement: Additional file 5: Figure S1. — MDS plot showing relationships of RNA-seq samples. (PDF 31 kb) [file 12864_2016_2395_MOESM5_ESM.pdf]

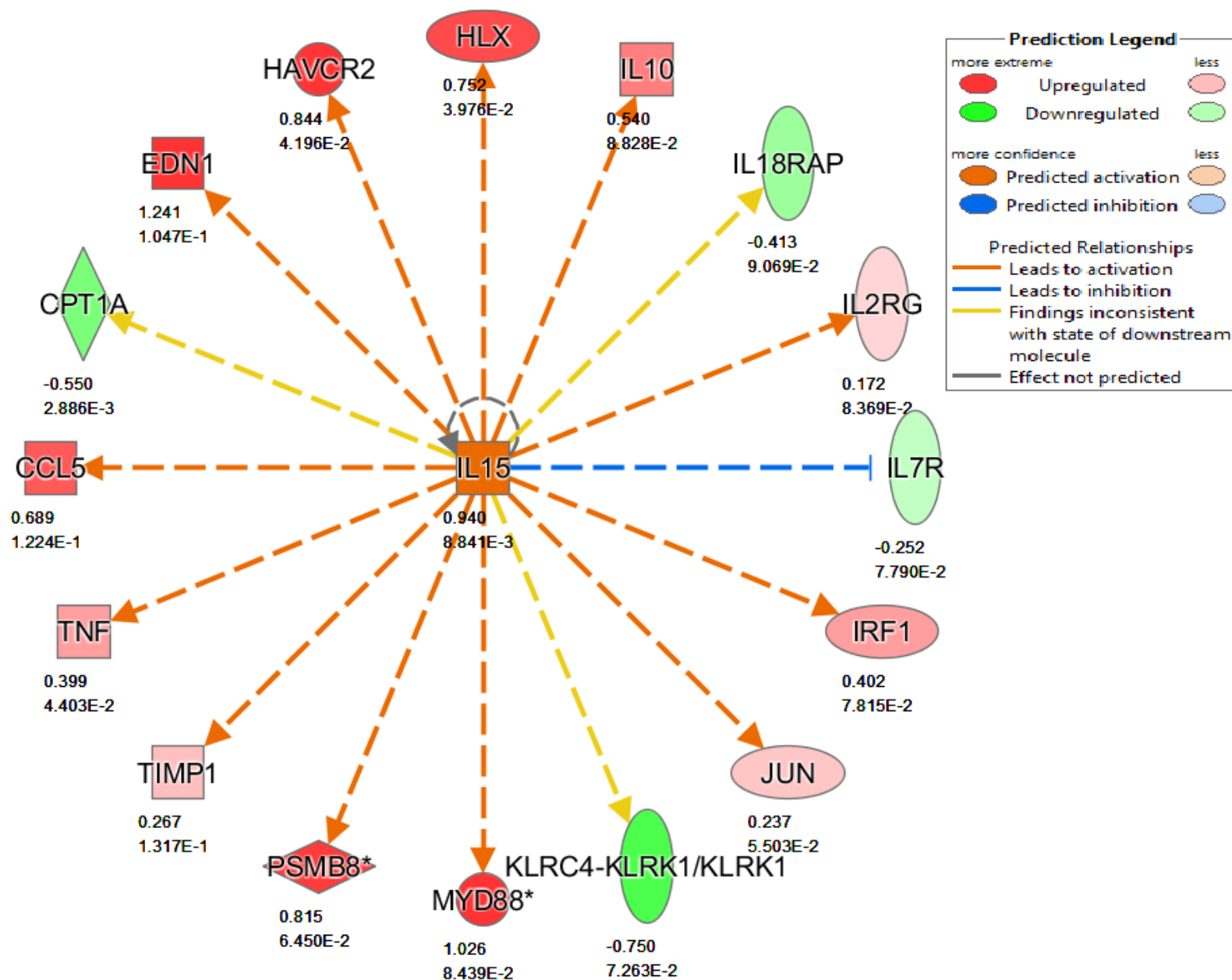

Supplement: Additional file 7: Figure S2. — Subnetwork consisting of differentially expressed genes regulated by the inferred upstream regulator IL-15. Nodes were colored based on the log ratio of averaged expression levels of genes between groups, with up-regulated genes in red and down-regulated genes in green. (PDF 86 kb) [file 12864_2016_2395_MOESM7_ESM.pdf]
